# Mining the Roles of Wheat (*Triticum aestivum*) SnRK Genes in Biotic and Abiotic Responses

**DOI:** 10.3389/fpls.2022.934226

**Published:** 2022-06-30

**Authors:** Baihui Jiang, Yike Liu, Hongli Niu, Yiqin He, Dongfang Ma, Yan Li

**Affiliations:** ^1^Engineering Research Center of Ecology and Agricultural Use of Wetland, Ministry of Education/College of Agriculture, Yangtze University, Jingzhou, China; ^2^Hubei Key Laboratory of Food Crop Germplasm and Genetic Improvement, Food Crops Institute, Hubei Academy of Agricultural Sciences/Wheat Disease Biology Research Station for Central China, Wuhan, China; ^3^Longgan Lake National Nature Reserve Authority of Hubei, Huanggang, China

**Keywords:** TaSnRK, biotic and abiotic stresses, qRT-PCR, TaSnRK2.4-B, negative regulator

## Abstract

Sucrose non-fermenting-1-related protein kinases (SnRKs) play vital roles in plant growth and stress responses. However, little is known about the SnRK functions in wheat. In this study, 149 TaSnRKs (wheat SnRKs) were identified and were divided into three subfamilies. A combination of public transcriptome data and real-time reverse transcription-polymerase chain reaction (qRT-PCR) analysis revealed the distinct expression patterns of TaSnRKs under various abiotic and biotic stresses. TaSnRK2.4-B, a member of SnRK2s, has different expression patterns under polyethylene glycol (PEG), sodium chloride (NaCl) treatment, and high concentrations of abscisic acid (ABA) application. Yeast two-hybrid assay indicated that TaSnRK2.4-B could interact with the SnRK2-interacting calcium sensor (SCS) in wheat and play a role in the ABA-dependent pathway. Moreover, TaSnRK2.4-B might be a negative regulator in wheat against pathogen infection. The present study provides valuable information for understanding the functions of the TaSnRK family and provides recommendations for future genetic improvement in wheat stress resistance.

## Introduction

Sucrose non-fermenting-1-related protein kinase (SnRK), a class of serine/threonine protein kinases, is widely present in the plant kingdom and strongly conserved among species. It modulates specific proteins to regulate the interrelationship of multiple signaling pathways using phosphorylation in plants and plays a vital role in their stress responses (Halford and Hey, 2009; Yan et al., 2014).

The SnRKs are subdivided into three subfamilies, SnRK1, SnRK2, and SnRK3, according to the structural characteristics of proteins (Hrabak et al., 2003; Coello et al., 2011). All plant SnRK subfamilies share a common S_TKc domain (Serine/Threonine protein kinases, PF00069). The SnRK1 subfamily specifically contains a C-terminal kinase-associated domain 1 (KA1 domain, PF02149) (Amodeo et al., 2007). The SnRK2 has an SnRK2-specific box (glutamine-303 to proline-318) (Christophe et al., 2006), and the NAF domain (Asn-Ala-Phe, PF03822) is present in the SnRK3 subfamily (Albrecht et al., 2001).

Concomitant with the diverse domains, the functions of the three subfamilies are different. In SnRK1, a heterotrimeric complex composed of α, β, and γ subunits, is homologous to the yeast SNF1 gene, which was first discovered and well-known for its role as an energy sensor in the global regulation of carbon metabolism (Hardie et al., 1998). Over-expression of MhSnRK1 in tomatoes could regulate fruit development and increase both the carbon and nitrogen assimilation rate (Wang et al., 2012). Both SnRK2 and SnRK3 are plant-specific and are involved in signaling pathways that regulate plant responses to osmotic and adversity stresses. There are compelling pieces of evidence indicating that the divergence of SnRK2 and SnRK3 subfamilies evolved after the duplication of SnRK1, which has enabled plants to form networks connecting metabolic and genetic responses to stress, hormone, and calcium signal (Halford and Hey, 2009).

The first identified SnRK2 gene is PKABA1 (accession no. M94726), which was isolated from wheat after ABA treatment and can be induced by ABA and drought treatment (Anderberg and Walker-Simmons, 1992). Among the ten SnRK2 genes in *Arabidopsis*, nine (except for SnRK2.9) can be activated by hyperosmotic and salinity stresses. Furthermore, five (SnRK2.2, SnRK2.3, SnRK2.6, SnRK2.7, and SnRK2.8) of the nine genes can be activated by ABA (Boudsocq et al., 2007; Coello et al., 2011). SnRK3 are also known as calcineurin B-like proteins (CBL) interacting protein kinase (CIPK), which interacts with calcium sensor CBL to transfer stress signals (Sheen, 1996; Albrecht et al., 2003). The best-studied member is salt overly sensitive 2 (SOS2), which is involved in response to salt stress and ABA signaling and is required for sodium ion (Na^+^) and potassium ion (K^+^) homeostasis and abiotic stress tolerance (Liu et al., 2000; Guo et al., 2001).

Bread wheat (*Triticum aestivum* L.) is one of the most important food crops worldwide. Several members of the TaSnRK family genes have been identified and their functions explored. TaSnRK1α plays an important role in Fusarium head blight (FHB) resistance and is the common target of TaFROG (*Fusarium* Resistance Orphan Gene), and *Fusarium graminearum* orphan secreted protein Osp24. These two orphan proteins bind with TaSnRK1α competitively during the conflict between wheat and *F. graminearum* (Perochon et al., 2015; Jiang et al., 2020). To date, ten TaSnRK2 genes have been isolated from wheat (Zhang et al., 2016). These genes are multifunctional regulatory factors that are involved in response to various abiotic stresses (Mao et al., 2010; Zhang et al., 2010; Feng et al., 2019; Miao et al., 2021), and in thousand-kernel weight, photosynthesis, and other physiological aspects of wheat (Tian et al., 2013; Miao et al., 2017, 2021). However, the role of TaSnRK2s in biotic stresses is limited. In this study, the genes belonging to the whole SnRK family were identified through the wheat genome. The expression patterns of *TaSnRKs* under biotic and abiotic stresses were subsequently characterized. Furthermore, TaSnRK2.4-B was found to be participated in the pathway of pathogen-associated molecular patterns (PAMP) triggered immunity in *Nicotiana benthamiana*. The results of the present study provide useful information for understanding the functions of the TaSnRK family and provide recommendations for the future genetic improvement of stress resistance in wheat.

## Materials and Methods

### Identification of TaSnRK Genes

The protein sequences of known 38 AtSnRKs and 47 OsSnRKs (Supplementary Material 1) were collected and used as a query for BLASTp (2.2.28) against the wheat reference genome IWGSC v1.1 (Hrabak et al., 2003; Saha et al., 2014; Wang et al., 2015). The samples were further screened using SMART (9.0) and NCBI CDD (v 3.18) to confirm conserved domains in the wheat SnRK family. In general, the S_TKc domain (Serine/Threonine protein kinases, catalytic domain) is present in all SnRK genes; KA1 is specifically present in SnRK1 subfamily (Amodeo et al., 2007); SnRK2-specific box (glutamine-303 to proline-318) is present in SnRK2 subfamily (Christophe et al., 2006); and, NAF (Asn-Ala-Phe) domain is specifically present in SnRK3 subfamily (Albrecht et al., 2001). According to these specific domains in different subfamilies, TaSnRKs were screened and classified. The characteristic features of TaSnRK proteins were predicted using ExPASy Server (v10). The subcellular localization of TaSnRKs was predicted using Plant-mPLoc (2.0). The information on the chromosomal location of TaSnRKs was extracted from the reference IWGSCv1.1 GFF3 file. MapInspect software was used to draw the physical map of TaSnRKs.

### Phylogenetic Analysis of Wheat SnRK Gene Family

To further explore the evolutionary relationships of wheat SnRKs, multiple sequence alignments of SnRK proteins were conducted with ClustalW (2.0.10). A phylogenetic tree was constructed using the neighbor-joining (NJ) method with 1000 replicated bootstraps in MEGA X. The midpoint rooted base tree was modified via iTOL online tool (v3.2.317).

### Gene Structure and Protein Motif Analysis

The gene exon-intron structures of TaSnRK genes were performed by GSDS (v2.0) (Hu et al., 2015). The conserved motifs of TaSnRK proteins were identified by MEME (v4.9.1). Finally, the motif patterns were exhibited by TBtools software (Chen et al., 2020).

### Cis-Elements Analysis and Expression Pattern Analysis of TaSnRKs

Cis-acting elements in the upstream sequences (1.5 kb) of TaSnRKs were predicted using PlantCARE online tool. The result was organized and displayed by the R package “pheatmap”. RNA-seq data was downloaded from expVIP (Ramírez-González et al., 2018). Morpheus was used to generate heatmaps of TaSnRKs fold change based on log2 (TPM+1) values. The TaSnRKs were classified according to their expression levels using k-means clustering (K-means = 15) (Jain, 2010).

### Plant Treatment and qRT-PCR Analysis

Twenty seeds of Yangmei were surface-sterilized and germinated at 20°C for 2 days and then placed in a half-strength Hoagland nutrient solution. The seedlings were treated with 150 mM NaCl, 20% PEG 6000, and 100 μM ABA at one heart and one leaf stage. Roots were sampled at 2, 12, 24, 48, and 72 hours after treatments. Meanwhile, the plants were inoculated with *F. graminearum* (strain PH-1) (Yang et al., 2010) and powdery mildew (*Blumeria graminis f. sp. Tritici*, Bgt strain E09). The inoculated spikelets and leaves were harvested at 1, 3, and 5 days post inoculation (dpi). All treatments were implemented with three biological replications.

Total RNA was isolated from 100 mg tissues using TRIzol reagent (Invitrogen, USA) and was digested with DNase I (TaKaRa, Beijing, China) to eliminate genome DNA. Using RevertAid Reverse Transcriptase kit (Vazyme, Nanjing, China), cDNA was synthesized. Nine genes were selected, and the primers were listed in Supplementary Material 2. Then, qRT-PCR was performed in a 15 μl volume containing 2× SYBR Green qPCR Master Mix (Vazyme, Nanjing, China). The relative gene expression level was calculated with the 2^−ΔΔCt^ method (Livak and Schmittgen, 2001).

### Subcellular Localization and Transient Overexpression of TaSnRK2.4-B

The CDS region of TaSnRK2.4-B was cloned and inserted into the upstream green fluorescent protein (GFP) in the plant expression vector pART27. Then the recombinant vector was transformed into *Agrobacterium tumefaciens* strain GV3101, and the bacterial solution was injected into the leaves of 4–6-week-old *Nicotiana benthamiana*. For subcellular localization assay, 48 h post injection, the green fluorescence signal was detected and captured by laser scanning confocal microscopy (OLYMPUS, FV3000, Japan). For the luminol-based assay, 48 h post injection, 12 leaf disks were collected after washing with distilled water and were placed in the dark for 2 h. The leaf disks were then placed in an ELISA plate with 200 μl distilled water overnight in the dark. Later, the distilled water was replaced with luminol and horse radish peroxidase (HRP) solution. Finally, 50 μl 5× flg22 solution was added, and the photochemical signals were monitored immediately with a microplate reader (Hidex, SENSE 425-301, Finland).

### Yeast Two-Hybrid Assay

To detect the interactions between TaSnRK2.4-B and SnRK2-interacting calcium sensor (SCS) in wheat, the coding sequence of TaSnRK2.4-B was cloned into pGBKT7, and the coding sequence of SCS in wheat was cloned into pGADT7. The primers are listed in Supplementary Material 2. The recombinant vectors were transformed into yeast strain AH109. Afterward, the positive clones were confirmed on SD medium without Trp, Leu, His, and Ade according to the manufacturer's instruction.

## Results

### Identification and Phylogeny Analysis of TaSnRK Genes

**Figure 1:**
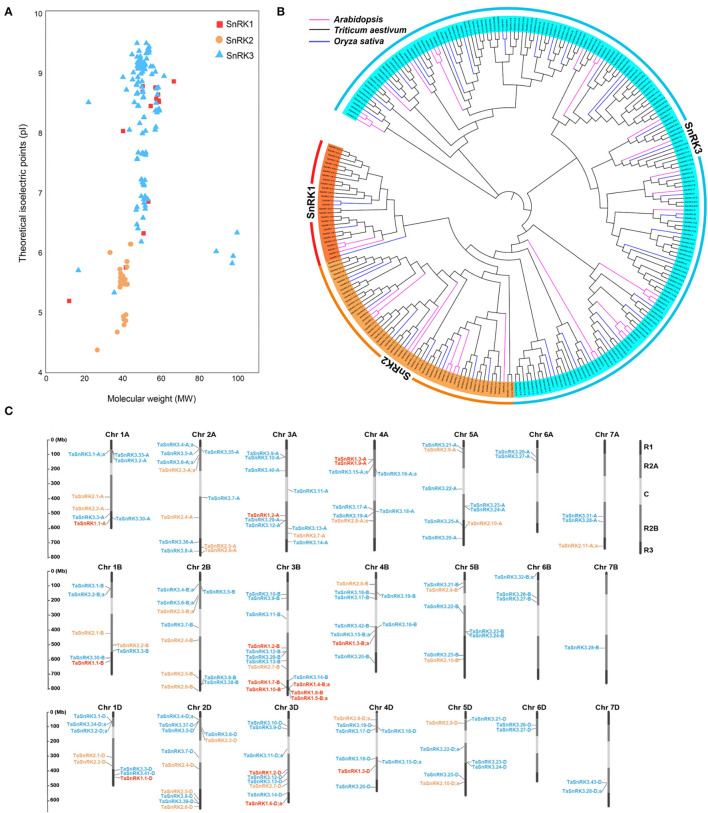
General information of TaSnRKs. **(A)** The distribution patterns of isoelectric points (pI) and molecular weights (MW) for the three subfamilies of wheat SnRKs. **(B)** Phylogenetic tree of non-fermenting-1-related protein kinase (SnRK) predicted in wheat and those previously identified in *Arabidopsis* and *Oryza sativa*. All amino acid sequences, including 186 TaSnRKs, 47 OsSnRKs, and 38 AtSnRKs, were aligned using ClustalW2, and the phylogenetic tree was constructed using the bootstrap neighbor-joining method (NJ) tree (1,000 replicates) method and MEGA 7.0 software. Different color ribbons mark different sub-families. SnRKs from wheat, rice, and *Arabidopsis* are distinguished with different color lines. **(C)** Chromosomal locations of the 148 TaSnRK genes. The ruler line on the left indicates the physical map distance among genes (Mbp). Dark black represents R1 and R3 (distal telomeric parts of the chromosomes), light gray represents R2A and R2B (sub-central segments of the chromosomes), and white represents C (central segments of the chromosomes). Gene members of SnRK1 (red), SnRK2 (orange), and SnRK3 (blue) were mapped to corresponding chromosomes. TaSnRK3.44-U gene was not mapped in chromosomes because it was not predicted in any chromosomes. The specific location information of each gene on the chromosomes is listed in Supplementary Material 2.

After querying BLASTp with 38 AtSnRK and 47 OsSnRK proteins, 1,240 putative TaSnRK proteins were found. However, after conserved domain searching by NCBI CDD and SMART tools, 149 genes (186 proteins due to the alternative splicing) were finally retained. It contained 21 SnRK1 proteins, 38 SnRK2 proteins, and 127 SnRK3 proteins (Figure 1A; Supplementary Material 3). The wheat SnRKs were named according to their subfamily (SnRK1, SnRK2, and SnRK3) and genome location (Supplementary Material 3). Triplicate genes share the same gene number but use suffixes A, B, and D to distinguish sub genome location. Consecutive lowercases were separated by a semicolon to distinguish the alternative splicing variants (e.g., TaSnRK1.3-B;a and TaSnRK1.3-B;b) (Ramírez-González et al., 2018).

To further understand the features of TaSnRKs, the protein properties of 186 TaSnRK proteins were analyzed (Supplementary Material 3). The length of TaSnRKs proteins ranged from 103 (TaSnRK1.10-B) to 885 (TaSnRK3.32-B;c) amino acids (aa). The average molecular weight (MW) of SnRK1, SnRK2, and SnRK3 subfamily proteins were 53, 40, and 51 kDa, respectively. And the theoretical isoelectric point (pI) ranged from 4.38 (TaSnRK2.8-D;a) to 9.51 (TaSnRK3.12-B and TaSnRK3.21-B). As shown in Figure 1A, compared to the other two subfamilies, SnRK2 had lower MW values, and all pI values were less than 7. All the TaSnRKs belonged to hydrophilic proteins as their grand average of hydropathicity (GRAVY) values were negative.

The phylogenetic tree generated by SnRK protein sequences from Arabidopsis, rice, and *T. aestivum* showed that TaSnRKs belonging to the same subfamilies were clustered together (Figure 1B). Moreover, members of the SnRK2 subfamily had a closer relationship with those in SnRK1.

As illustrated in Figure 1C, TaSnRK genes were located on 21 chromosomes, except for TaSnRK3.44-U in an unanchored contig (Supplementary Material 3). Moreover, the chromosome distributions were uneven. Compared to gene numbers in other chromosomes, fewer genes were located in Chr 6 and Chr 7. Also, more TaSnRKs (73.3%) were located in the central segments (R2a, R2b, and C) of the chromosomes (Ramírez-González et al., 2018).

### Motif Composition, Exon-Intron Structure, Protein Feature, and Structure Analysis

**Figure 2:**
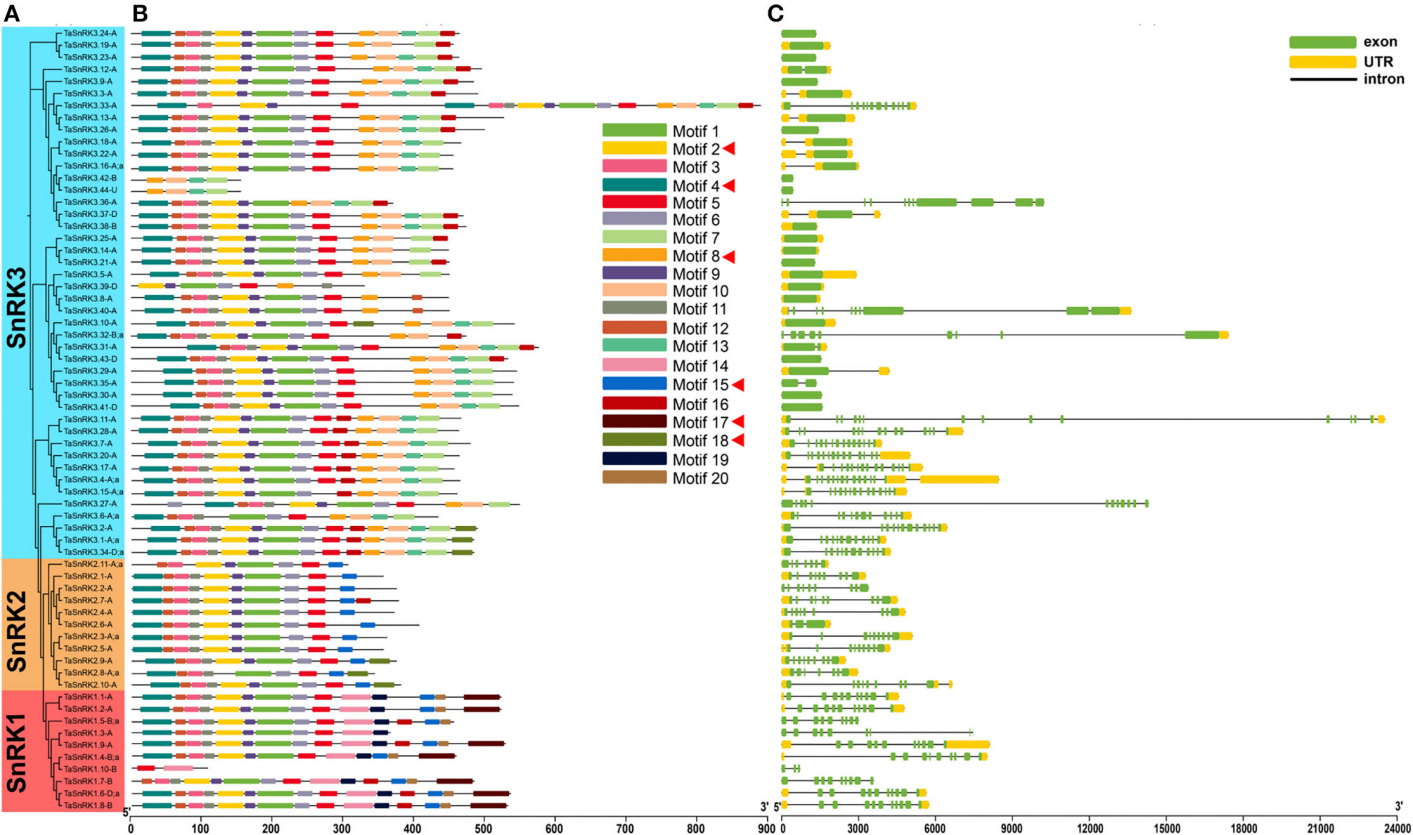
Motif composition and exon-intron structure of 65 representative TaSnRKs. **(A)** Phylogenetic tree of TaSnRKs was created by MEGA X with the neighbor-joining (NJ) method. **(B)** Motif composition of TaSnRKs. Different motifs are indicated by a specific color. The red triangle indicates motifs related to the functional domains. **(C)** Gene structure of TaSnRKs. Gene structure and motif analyses of 186 TaSnRKs can be found in Supplementary Material 4.

TaSnRKs were analyzed according to the following criteria: (1) one gene of the triplicates was retained; (2) only the first variant was kept. Consequently, 65 typical TaSnRKs were selected. As illustrated in Figures 2A,B and Supplementary Material 4, the proteins with a close relationship in the phylogenetic tree shared similar motif compositions. There were six conserved motifs related to the functional domains (Supplementary Material 5). Motif 2 and motif 4 contained Ser/Thr active site and ATP-binding region, respectively. Motif 17 contained the specific conserved KA1 domain of SnRK1, and motif 8 contained the specific NAF domain of SnRK3. Furthermore, motif 18, present in some members of the SnRK2 and SnRK3 subfamilies, contained an ABA-specific box, which indicated that these members might play roles in response to ABA stress. As shown in Figure 2C, all genes in SnRK1 and SnRK2 subfamilies had introns. On the contrary, 40% of SnRK3 genes had no intron. The differences in gene and protein structure among the three subfamilies illustrate the possibility of gene subfunctionalization or neofunctionalization in the TaSnRKs family.

### Promoter Analysis

**Figure 3:**
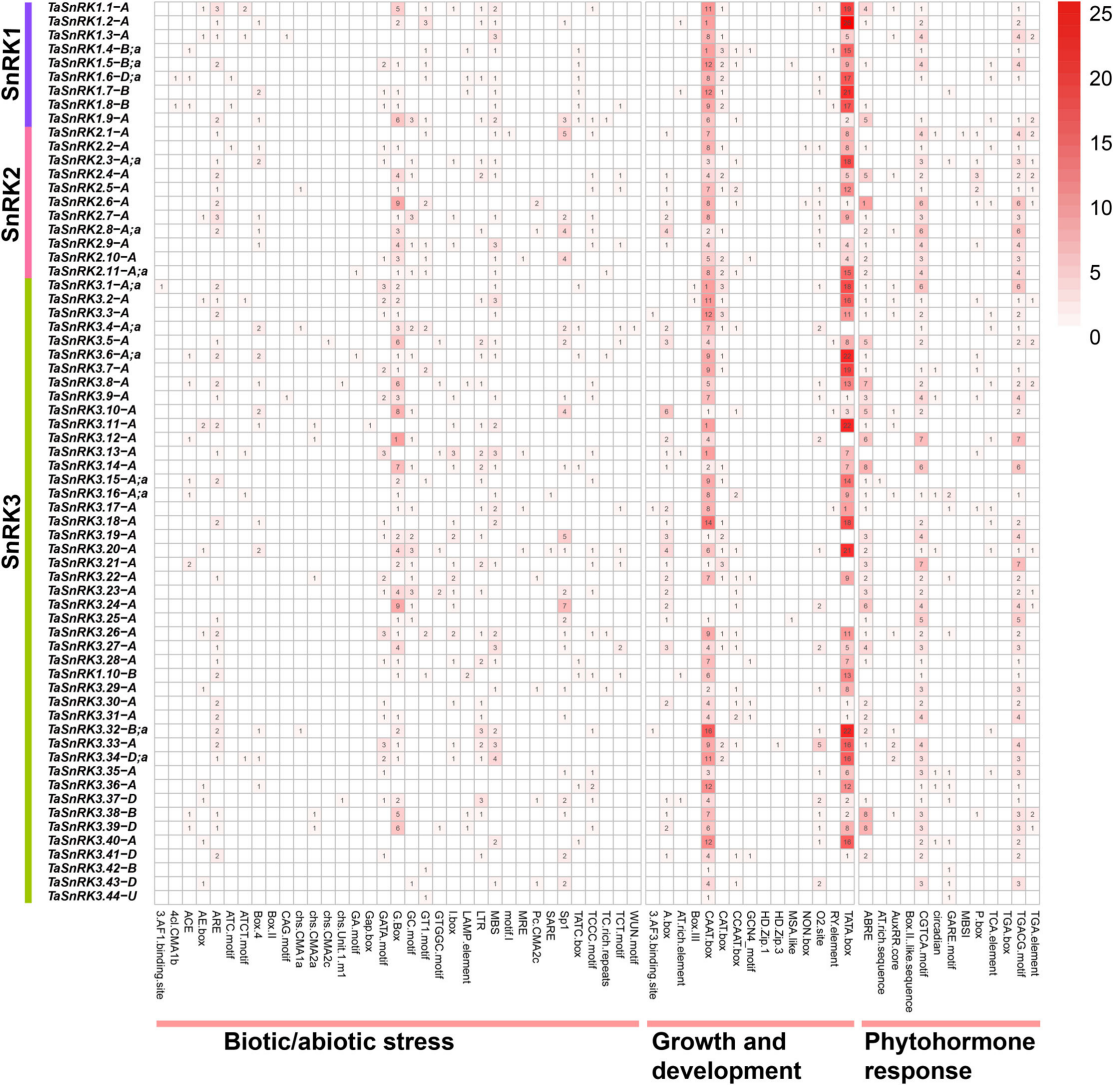
Distribution and summarizing of predicted cis-acting elements in the 1.5 kb upstream of 65 representative TaSnRK genes. Cis-acting elements involved in biotic/abiotic stresses, growth and development, and phytohormone response in the promoter of the 65 TaSnRK genes. The different numbers and colors suggest the elements' numbers. All cis-acting elements of 186 TaSnRKs promoter are displayed in Supplementary Material 6.

The sequence analysis of 1.5 kb upstream of TaSnRK genes showed that the cis-acting elements were clustered into three categories, and most of the cis-acting elements were related to growth and development (Figure 3, Supplementary Material 6). CAAT-box, a common element in enhancer and promoter areas that plays an essential role in transcription, is predominant among these genes (98.5%). Other cis-acting elements, such as A-box, AT-rich sequence, CAT-box, CCAAT-box, GCN4_motif, and O_2_-site, which participate in multiple growth regulation processes, were also found in most of the genes. Furthermore, elements related to hormone signaling pathways, like auxin (AuxRR-core and TGA-element), abscisic acid (ABRE), gibberellin (TATC-box, GARE-motif, and P-box), salicylic acid (TCA-element and SARE), and methyl jasmonate (CGTCA-motif and TGACG-motif), were present in the promoter regions of TaSnRKs.

### Expression Patterns of TaSnRKs in Temporal and Spatial Variation and Different Stress Conditions

**Figure 4:**
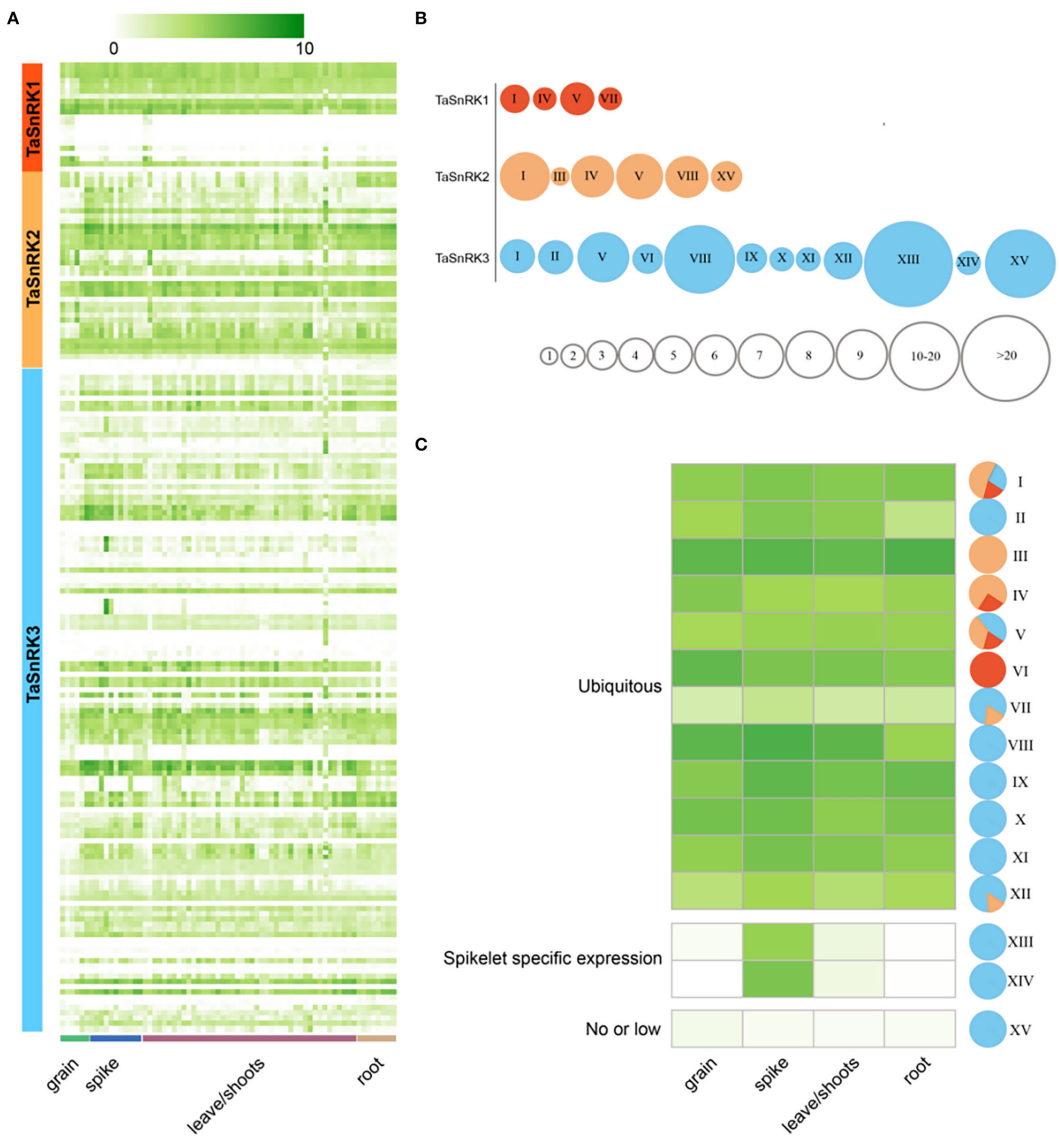
Transcriptome analyses of 186 TaSnRKs during wheat development. **(A)** The 186 TaSnRK gene expression levels in three wheat development stages/tissues (columns) and subfamilies (rows). Different levels of gene fold change, i.e., log2 (TPM+1), are marked by colors that gradually change (Fold change > 5 suggests significant expression). Detailed information is listed in Supplementary Material 7. **(B)** Based on the gene expression, genes in three subfamilies were clustered into 15 types indicated by roman numerals. Smaller circles indicate fewer genes of this subfamily belong to this type (a particular circle size suggests gene numbers). **(C)** A heatmap shows average expression values for the 15 types. Colors in sectors indicate three subfamilies as **(A,B)**: SnRK1 (red), SnRK2 (orange), and SnRK3 (blue).

To determine the expression patterns of TaSnRKs in different tissues during different stages, 209 RNA-seq data of Azhurnaya (a hexaploid wheat variety) were analyzed (Figure 4A; Supplementary Material 7, 8) (Ramírez-González et al., 2018). Of these, 18.81% (6 SnRK1s genes; 3 SnRK2s genes; and 26 SnRK3s genes) were not expressed due to the expression level TPM < 1. As shown in Figure 4A, there was no tissue-specific gene among TaSnRKs. The TaSnRKs were clustered into 15 modules according to similarity of expression patterns (Figure 4B). There were only four modules in SnRK1, while there were 12 modules in the SnRK3 subfamily. There were genes from all three subfamilies exhibited in modules I and V, showing the universality of these two modules. In general, the genes from TaSnRK1 and TaSnRK2 subfamily were ubiquitously expressed during the whole plant life (Figure 4C). In addition to being generally expressed (39/127, 30.7%), the TaSnRK3 genes were also highly expressed in reproductive organ spikelets [module XIII (66/127, 60.0%) and XIV (2/127, 1.6%)], which indicated that these genes might play roles during the development of spikelet. Interestingly, most of the low or not-expressed genes (module XV) belonged to the SnRK3 subfamily (20/127, 15.7%).

**Figure 5:**
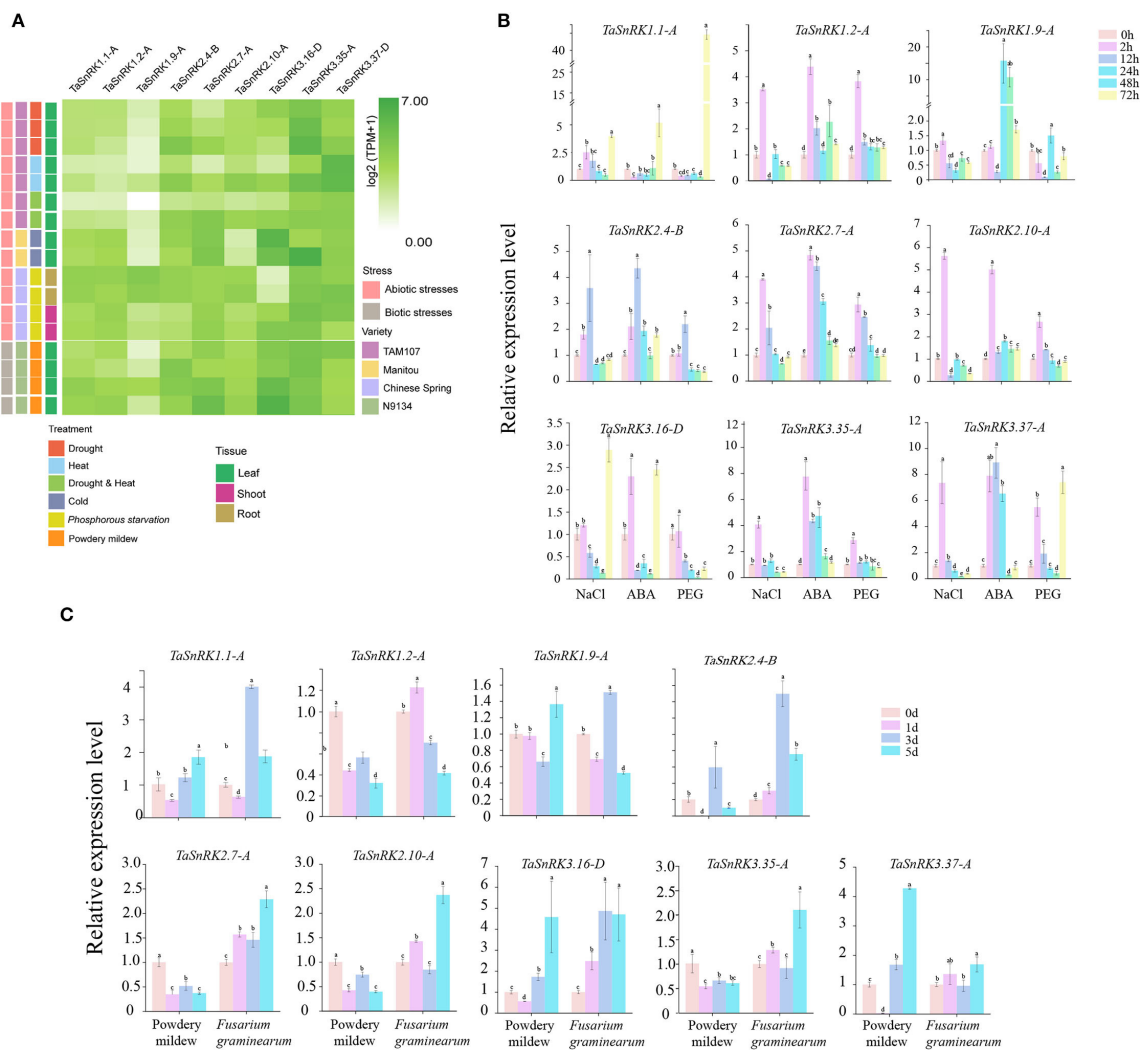
Expression patterns of nine selected TaSnRK genes under abiotic and biotic stresses. **(A)** Expression levels of nine TaSnRK genes under multiple abiotic stresses involving PEG 6000 stress, cold stress, drought and heat combined stress, drought stress, heat stress, phosphorous starvation, and biotic stress involving powdery mildew infection. **(B)** Expression patterns of the nine TaSnRK genes under abiotic stresses including NaCl, ABA, and PEG treatment. **(C)** Expression of the nine TaSnRK genes under biotic stresses, including powdery mildew and *Fusarium graminearum* infection. The relative expression levels were determined by the 2^−ΔΔct^ method. Significant differences at *P* < 0.05.

To examine the responses of TaSnRKs to abiotic and biotic stresses, nine genes belonging to the three subfamilies with universal expression patterns were randomly selected (Figure 5A). After cold treatment for 2 weeks, the expression level of TaSnRK2.4-B was decreased. The expression pattern of TaSnRK3.16-D was similar to that of TaSnRK2.4-B. In contrast, the expression level of TaSnRK3.35-A was upregulated sharply. Under drought treatment, the expression levels of TaSnRK2.4-B, TaSnRK2.7-A, and TaSnRK3.35-A were increased significantly, which indicated that these genes might play positive roles against drought stress. The expression patterns after NaCl and ABA treatment were detected by qRT-PCR. As illustrated in Figure 5B, most genes were induced at 2 hours after NaCl and ABA treatment. In contrast, the expression level of TaSnRK1.1-A declined after ABA treatment.

After inoculating with powdery mildew (Figure 5A), the expression levels of TaSnRK2.4-B, TaSnRK2.7-A, and TaSnRK3.16-D were significantly up-regulated. In contrast, the expression level of TaSnRK3.37-D declined with infestation time. However, in qRT-PCR results (Figure 5C), all the genes were unexpressed on the first day after powdery mildew infection, especially TaSnRK2.4-B and TaSnRK3.37-A, of which the expression level was decreased sharply. After being inoculated with *F. graminearum*, TaSnRK1.1-A, TaSnRK2.4-B, and TaSnRK3.16-D were significantly induced on the 3rd day. Consequently, these results indicate that TaSnRKs play different roles during various treatments.

### Subcellular Localization of TaSnRK2.4-B

**Figure 6:**
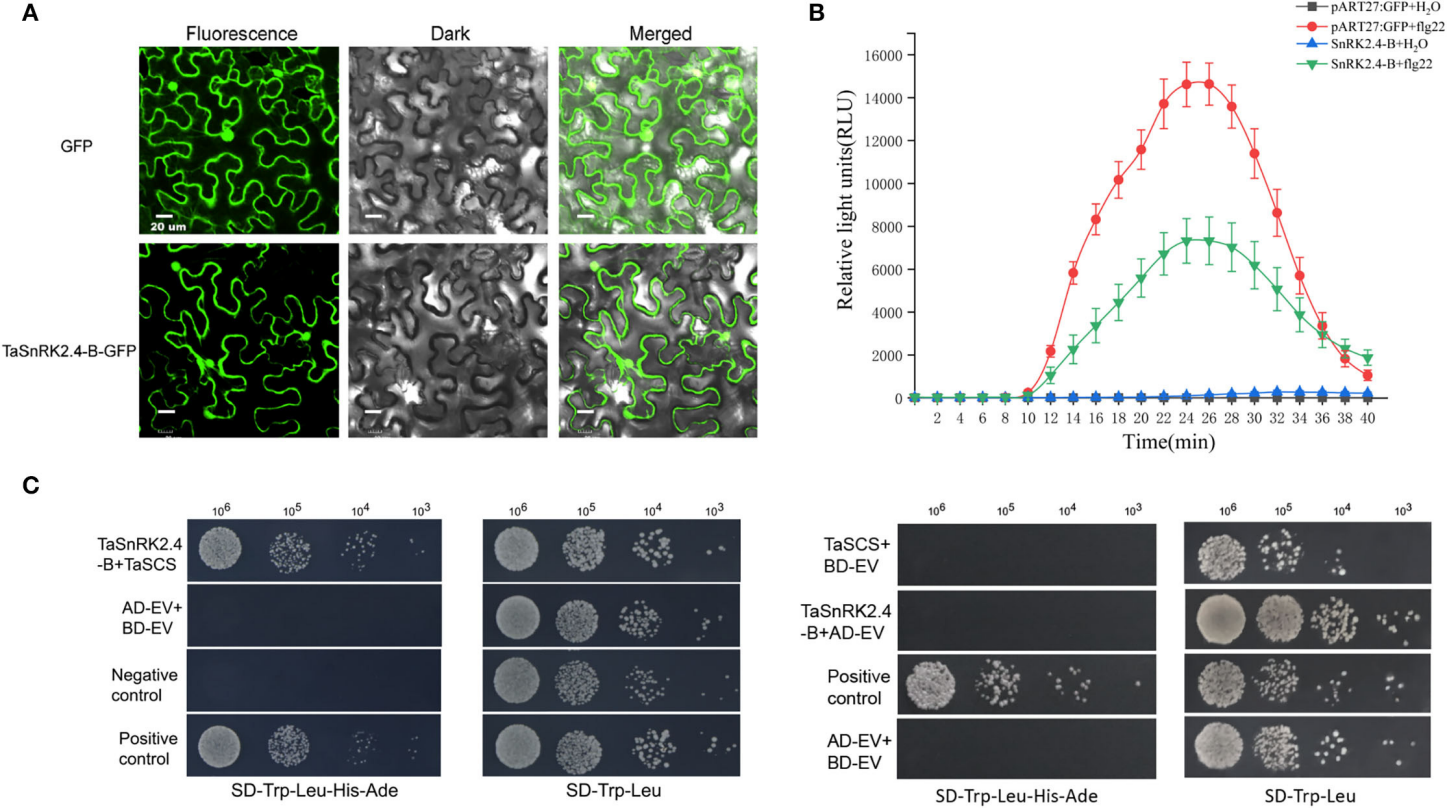
Functional analysis of TaSnRK2.4-B. **(A)** Subcellular localization of TaSnRK2.4-B. GFP fluorescence was visualized using confocal laser scanning microscopy. Scale bar = 20 μm. **(B)** Oxidative burst triggered by 5× flg22 solution in transient overexpression of TaSnRK2.4-B leaves and vector control measured in a luminol-based assay as relative light units. Values are averages ±SE (*n* = 12). **(C)** Different concentrations (cells/ml) of yeast transformants expressing the indicated bait and prey constructs were assayed for growth on SD-Trp-Leu and SD-Trp-Leu-His-Ade plates.

Subcellular localization of proteins could provide clues for further functional analysis. From the previous result, TaSnRK2.4-B was predicted to be localized in the nucleus (Supplementary Material 2). To validate the subcellular localization of TaSnRK2.4-B, the pART27-35S::TaSnRK2.4-B-GFP expression vector was successfully constructed and transferred into Agrobacterium GV3101. After transient expression in *N. benthamiana* leaves, the fluorescence signals of TaSnRK2.4-B-GFP was detected in the whole cell (Figure 6A), which was similar to the signal of blank control. This result was consistent with the localization results of its homologous genes (Zhang et al., 2016).

### Transient Overexpression of TaSnRK2.4-B Reduced the ROS Level Induced by flg22

As illustrated in Figure 6B, the overall temporal pattern of the reactive oxygen species (ROS) burst was not affected in the transiently overexpressed leaves of TaSnRK2.4-B. Similar to vector control, the ROS level in the transiently overexpressed leaves of TaSnRK2.4-B reached its peak at 26 mins after flg22 treatment. However, the total ROS produced by transient overexpression of TaSnRK2.4-B was only 50% of that of control. This result indicates that overexpression of TaSnRK2.4-B reduced the sensitivity of *N. benthinaniana* leaves against flg22 application.

### TaSnRK2.4 Can Interact With TaSCS

In Arabidopsis, AtSCS could interact with AtSnRK2 and inhibit AtSnRK2 activity (Bucholc et al., 2011; Tarnowski et al., 2019). Therefore, the interaction between TaSnRK2.4-B and TaSCS was tested by a yeast two-hybrid assay. There are 17 SCS homologous genes in wheat, and one of the closest homologs of AtSCS-A (TraesCS2D03G0940300.1) was selected (Supplementary Material 9). The Y2H result further confirmed that TaSnRK2.4-B could interact with TaSCS physically (Figure 6C).

## Discussion

SnRKs are highly conserved in eukaryotes and play important roles in plant development and response to various abiotic and biotic stresses (Tsai and Gazzarrini, 2012; Perochon et al., 2015; Nukarinen et al., 2016). In the present study, 149 non-redundant TaSnRK genes were identified (Figure 1; Supplementary Material 2). The number of TaSnRKs was 3.92 and 3.17 times higher than that in *Arabidopsis* and rice, respectively, which could be because of its hexaploidy and fragment replication (Mishra et al., 2021). A previous study showed that 94 proteins from 30 genes of the TaSnRK3 subfamily were identified (Sun et al., 2015). Our present study matches all these genes except TaCIPK6 because we did not retrieve its gene or CDS sequence from wheat genome data. The remaining 29 genes belonging to the SnRK3 subfamily were firstly identified in our result (Figure 1, Supplementary Material 2).

In previous studies, the SnRK2 family was sub-divided into three groups based on the expression pattern under ABA treatment. The members in Group I were not activated by ABA, and Group II SnRK2s responded weakly to ABA, whereas SnRK2s in Group III were strongly induced by ABA treatment (Zhang et al., 2016). Interestingly, we found that TaSnRK2.9-A, TaSnRK2.8-A;a, and TaSnRK2.10-A (namely TaSnRK2.9, TaSnRK2.10, and TaSnRK2.8 of Group III in Zhang's report) contained an ABA-specific box in protein (Figure 2B), which further proved that these three genes might play important roles in ABA signaling pathway. TaSnRK2.7 (renamed as TaSnRK2.4-D in this study), in Group I, showed no response to ABA (5 μM) (Zhang et al., 2011). However, our results showed that TaSnRK2.4-B, a triplicate gene of TaSnRK2.4-D, was significantly induced under 100 μM ABA (Figure 6B). Moreover, the expression level of TaSnRK2.7-A, which belonged to the same group I, was also sharply up-regulated and retained at the high level. For the inconsistent result, we speculated that it might be caused by different concentrations of ABA, and the genes in Group I could be induced by a high concentration of ABA.

**Figure 7:**
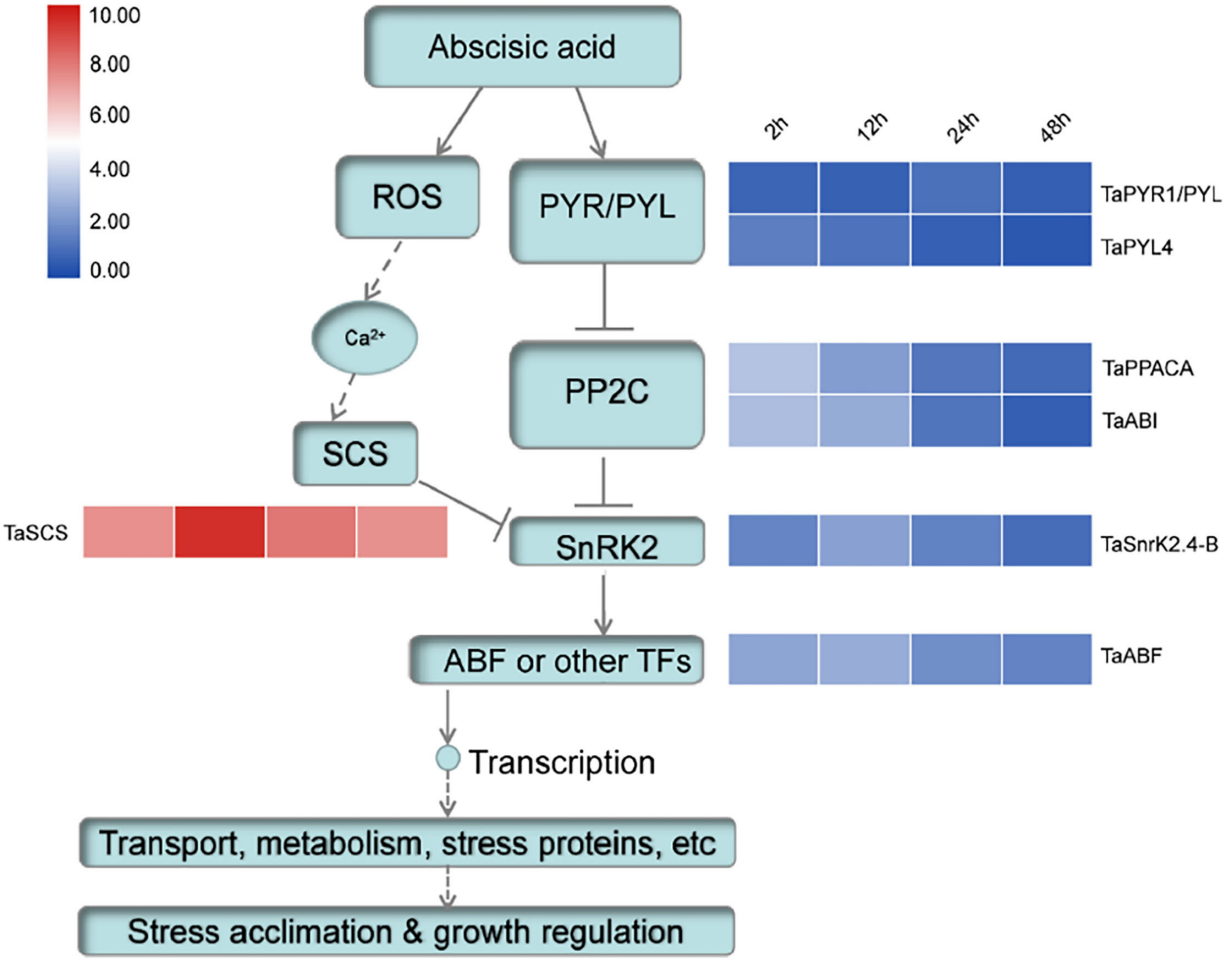
Expression level detected by qRT-PCR of key point genes involving ABA pathway in wheat.

Phytohormone ABA plays a significant role in plant growth and development and adaption to various stresses. SnRK2s are key positive regulators in ABA signaling (Nakashima and Yamaguchi-Shinozaki, 2013). In *Arabidopsis*, under stress conditions, ABA could activate the receptor PYR/PYL to interact with PP2Cs, which was binding with SnRK2s. Consequently, the ABA-dependent SnRK2s were released and play important regulatory roles in stomatal closure, growth, and stress response (Fujii and Zhu, 2009; Park et al., 2009). For equilibration, other proteins interact with SnRK2s to modify their activities. Previous studies showed that both AtSCS forms (AtSCS-A and AtSCS-B) could interact with SnRK2 and inhibit its activity in calcium-dependent and independent pathways (Bucholc et al., 2011; Tarnowski et al., 2019). To detect whether TaSnRK2 could interact with TaSCS, a Y2H assay was conducted. Figure 6C demonstrates that the TaSCS could interact with TaSnRK2.4-B. Furthermore, the expression patterns of several key genes in the ABA signaling pathway were detected. As shown in Figure 7, after ABA treatment, the expression level of TaPYR4 (homologous to AtPYR4) and TaPP2C (homologous to AtPP2CA and AtABI2) was continuously declined with stress time. However, expression levels of TaSnRK2.4-B and TaABF (homologous to AtABF) were increased at 12 hours. At the same time, the expression pattern of TaSCS (homologous to AtSCS-A) was consistent with that of TaSnRK2.4-B. These results indicated that the regulatory network in wheat was similar to that in *Arabidopsis*. Therefore, TaSCS probably inhibit the activity of TaSnRK2.4-B in wheat. This deduction needs further confirmation.

In contrast to the responses of TaSnRKs to abiotic stresses, the expression of all the tested genes was unexpressed on the 1st day after powdery mildew infection, especially that of TaSnRK2.4-B and TaSnRK3.37-A. This indicated that these genes may play negative roles against powdery mildew infection. To further explore the role of TaSnRK2.4-B in disease resistance, we detected the ROS burst in the *N. benthinaniana* leaves with transient overexpression of TaSnRK2.4-B after flg22 application. Flg22 is one of the typical pathogen-associated molecular patterns (PAMP) (Jones and Dangl, 2006). The plant immune system has two branches (PTI and ETI) to resist pathogen infection (Chisholm et al., 2006; Jones and Dangl, 2006). During the early stages of interaction, the plant produces a large number of reactive oxide species (ROS) to damage and inhibit pathogen invasion (Yoshioka et al., 2009). In the present study, the total ROS produced by transient overexpression of TaSnRK2.4-B was only 50% of that of control (Figure 6B). This result indicated that TaSnRK2.4-B participated in the PTI pathway. Moreover, the result demonstrated that TaSnRK2.4-B might be a negative regulator in disease resistance, which can be used to improve wheat through CRISPR technology.

## Conclusion

Wheat SnRK family genes were identified and classified into three sub-families. Transcriptomic profiling and qRT-PCR analyses indicated that the TaSnRKs played the role of multifunctional regulators responding to diversified biotic and abiotic stresses. Subcellular localization indicated that TaSnRK2.4-B existed in the whole cell. Through qRT-PCR analysis, TaSnRK2.4-B was induced after PEG, NaCl, and high concentration ABA treatment. Furthermore, it was found that TaSnRK2.4-B could interact with TaSCS and play roles in the ABA-dependent pathway. Moreover, TaSnRK2.4-B participated in the PTI pathway and might be a negative regulator in disease resistance.

## Data Availability Statement

The original contributions presented in the study are included in the article/Supplementary Material, further inquiries can be directed to the corresponding author/s.

## Author Contributions

YLi and DM designed this article. BJ, YLiu, HN, and YH directed the data analysis and manuscript writing. YLiu supervised the experiment and confirmed the manuscript. All authors contributed to the article and agreed to submit this version of the manuscript.

## Funding

This research was supported by the Open Program of Engineering Research Center of Ecology and Agricultural Use of Wetland Ministry of Education (KFT202103), the Open Project Program of Key Laboratory of Integrated Pest Management on Crop in Central China, and Ministry of Agriculture/Hubei Province Key Laboratory for Control of Crop Diseases, Pest and Weeds (2020ZTSJJ8).

## Conflict of Interest

The authors declare that the research was conducted in the absence of any commercial or financial relationships that could be construed as a potential conflict of interest.

## Publisher's Note

All claims expressed in this article are solely those of the authors and do not necessarily represent those of their affiliated organizations, or those of the publisher, the editors and the reviewers. Any product that may be evaluated in this article, or claim that may be made by its manufacturer, is not guaranteed or endorsed by the publisher.

## Supplementary Material

Supplementary Material 1 The query SnRK protein sequences of *Arabidopsis* and rice used in this study.

Supplementary Material 2 Primers used in this study.

Supplementary Material 3 Information including chromosomal location, protein features, subcellular localization, and source of 186 SnRK proteins identified in wheat.

Supplementary Material 4 Gene structure and motif patterns of all TaSnRKs.

Supplementary Material 5 All 20 MEME motif sequences in wheat SnRK proteins.

Supplementary Material 6 Cis-acting elements involved in stresses, growth and development, and phytohormone response in the promoter of 186 TaSnRKs.

Supplementary Material 7 Expression profile of wheat SnRKs from RNA-seq data.

Supplementary Material 8 The expression patterns of TaSnRKs in different tissues during different stages.

Supplementary Material 9 Phylogenetic tree of TaSCSs.
