# Supplementary figures and images for: Mining the Roles of Wheat (Triticum aestivum) SnRK Genes in Biotic and Abiotic Responses
Source: Front Plant Sci. 2022 Jun 30;13:934226. doi: 10.3389/fpls.2022.934226 (PMC9280681; doi:10.3389/fpls.2022.934226)

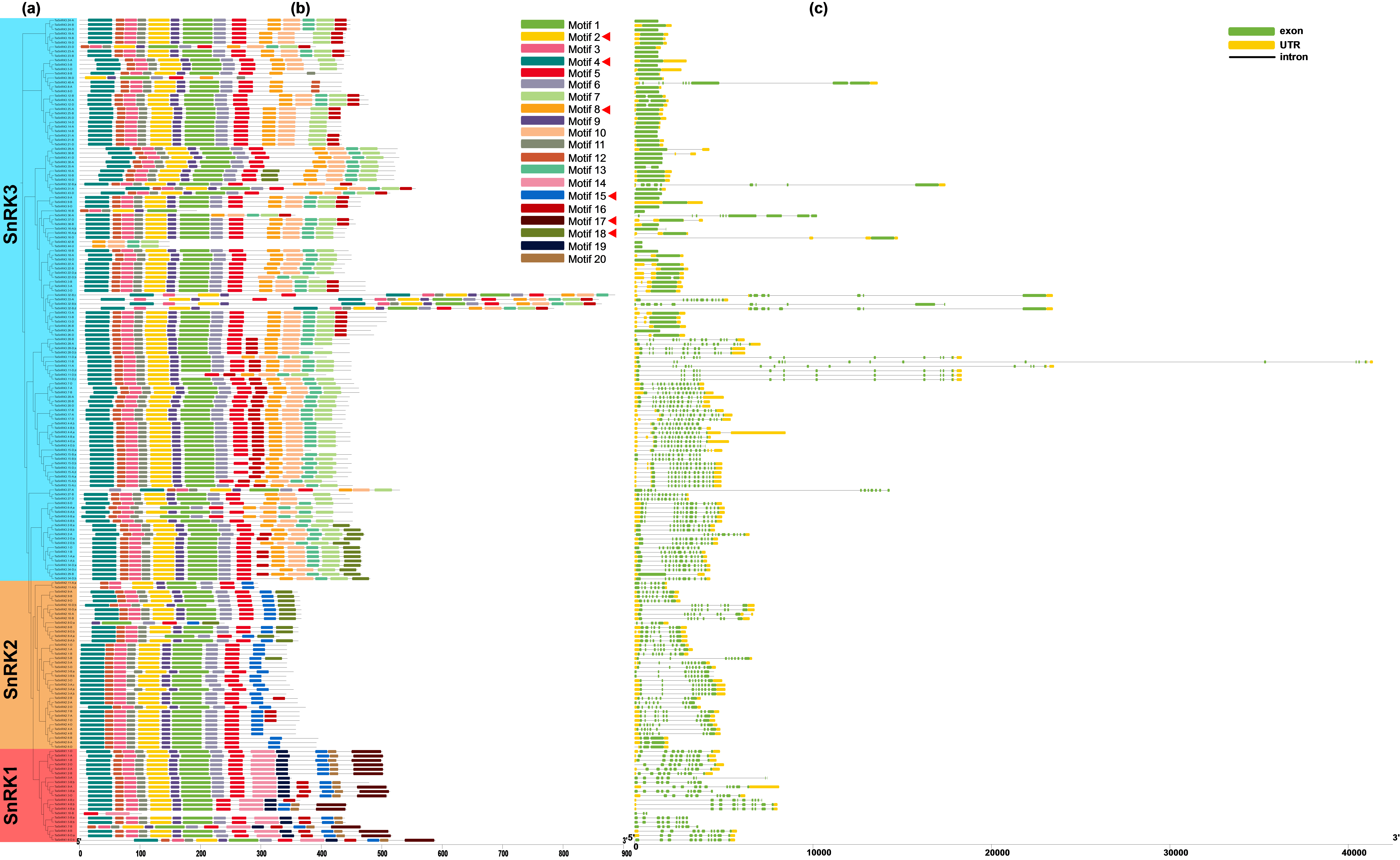

Supplement: Supplementary Material 4 — Gene structure and motif patterns of all TaSnRKs. [file Image_4.png]

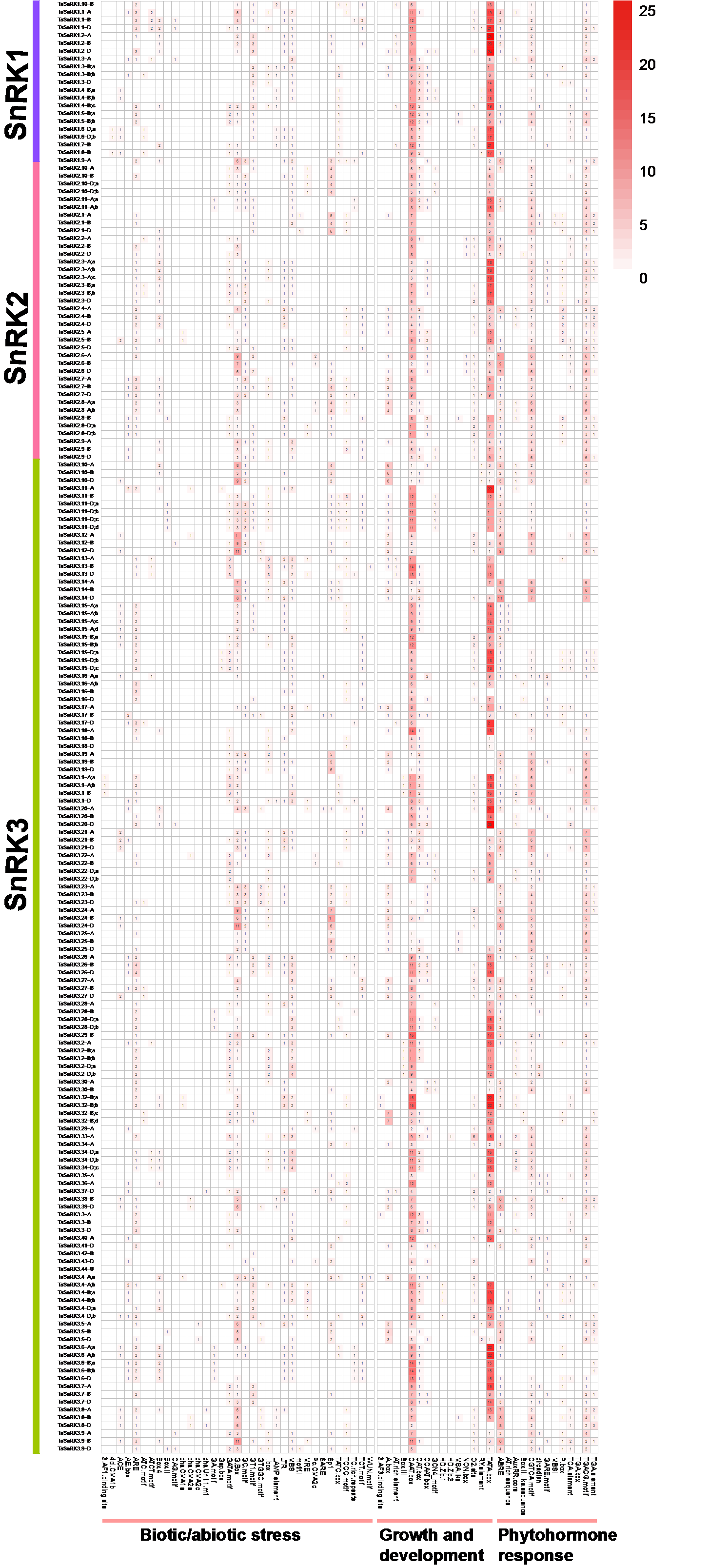

Supplement: Supplementary Material 6 — Cis-acting elements involved in stresses, growth and development, and phytohormone response in the promoter of 186 TaSnRKs. [file Image_6.png]

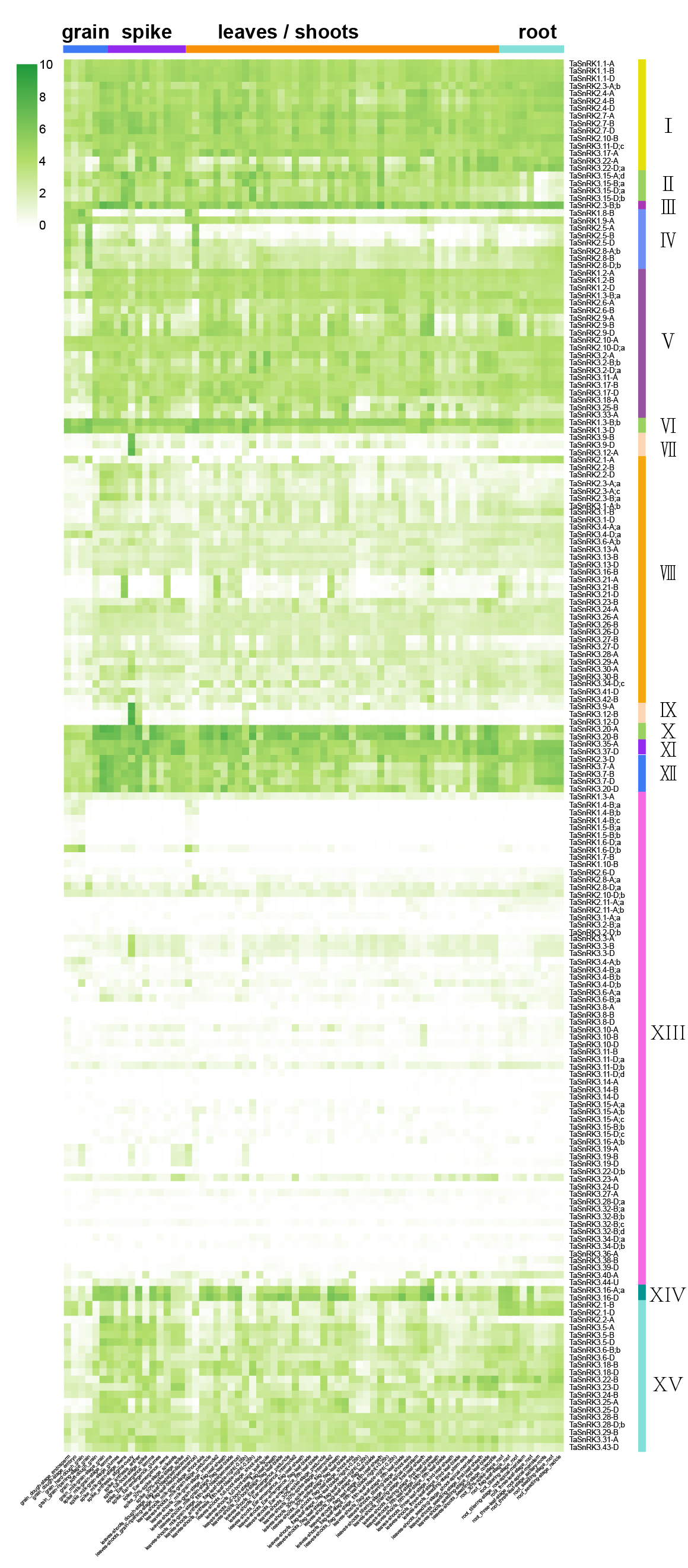

Supplement: Supplementary Material 8 — The expression patterns of TaSnRKs in different tissues during different stages. [file Image_8.png]

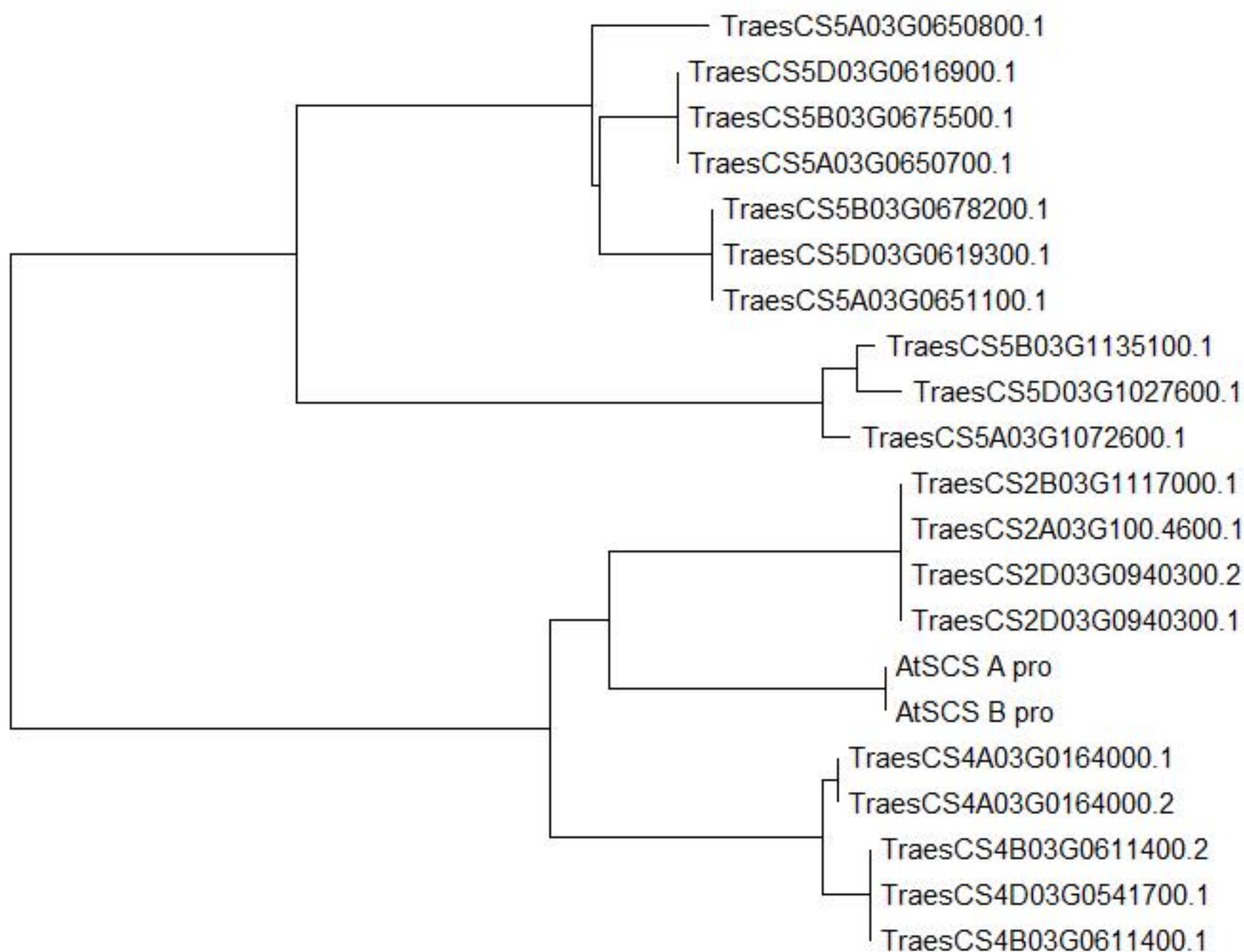

2

Supplement: Supplementary Material 9 — Phylogenetic tree of TaSCSs. [file Image_9.pdf]
